# Functional genome analysis and anti-*Helicobacter pylori* activity of a novel bacteriocinogenic *Lactococcus* sp. NH2-7C from Thai fermented pork (*Nham*)

**DOI:** 10.1038/s41598-023-47687-4

**Published:** 2023-11-21

**Authors:** Engkarat Kingkaew, Weerapong Woraprayote, Auttaporn Booncharoen, Kanidta Niwasabutra, Thitiphorn Janyaphisan, Ratha-Korn Vilaichone, Yoshio Yamaoka, Wonnop Visessanguan, Somboon Tanasupawat

**Affiliations:** 1 Department of Biology, School of Science, King Mongkut’s Institute of Technology Ladkrabang, Bangkok, 10520 Thailand; 2 Department of Biochemistry and Microbiology, Faculty of Pharmaceutical Sciences, Chulalongkorn University, Bangkok, 10330 Thailand; 3 National Center for Genetic Engineering and Biotechnology (BIOTEC), National Science and Technology Development Agency (NSTDA), Pathum Thani, 12120 Thailand; 4 Thailand Institute of Scientific and Technological Research (TISTR) Biodiversity Research Centre, Pathum Thani, 12120 Thailand; 5 GI Unit, Department of Medicine, and Center of Excellence in Digestive Diseases, Thammasat University, Thailand Science Research and Innovation Fundamental Fund, Bualuang ASEAN Chair Professorship at Thammasat University, Pathum Thani, 12120 Thailand; 6 Department of Environmental and Preventive Medicine, Faculty of Medicine Oita University, Yufu, Oita Japan

**Keywords:** Computational biology and bioinformatics, Immunology, Microbiology, Molecular biology, Diseases

## Abstract

*Helicobacter pylori*, linked to gastric diseases, is targeted for probiotic treatment through bacteriocin production. Bacteriocins have gained recognition for their non-toxic effects on host cells and their ability to combat a wide range of pathogens*.* This study aimed to taxonomically characterize and evaluate the safety and probiotic properties of the novel species of *Lactococcus* sp. NH2-7C isolated from fermented pork, as well as its bacteriocin NH2-7C, both in vitro and in silico. Comparative genotypic analysis revealed an average nucleotide identity of 94.96%, an average amino acid identity of 94.29%, and a digital DNA-DNA hybridization value of 63.80% when compared to *Lactococcus lactis* subsp. *lactis* JCM 5805^T^. These findings suggest that strain NH2-7C represents a novel species within the genus *Lactococcus*. In silico assessments confirmed the non-pathogenic nature of strain NH2-7C and the absence of genes associated with virulence and biogenic amine formation. Whole-genome analysis revealed the presence of the *nisA* gene responsible for nisin A production, indicating its potential as a beneficial compound with anti-*Helicobacter pylori* activity and non-toxic characteristics. Probiotic assessments indicated bile salt hydrolase and cholesterol assimilation activities, along with the modulation of interleukin-6 and tumour necrosis factor-α secretion. Strain NH2-7C demonstrated gastrointestinal tolerance and the ability to adhere to Caco-2 cells, affirming its safety and probiotic potential. Additionally, its ability to produce bacteriocins supports its suitability as a functional probiotic strain with therapeutic potential. However, further in vitro and in vivo investigations are crucial to ensure its safety and explore potential applications for *Lactococcus* sp. NH2-7C as a probiotic agent.

## Introduction

Lactic acid bacteria (LAB), including *Lactococcus* species commonly used as probiotics and starter cultures, have been extensively studied for their probiotic properties^1^. Probiotics, defined as beneficial live microorganisms when ingested adequately, include select LAB strains with key attributes like safety, and gastrointestinal persistence. These bacteria should also possess the ability to inhibit pathogens^2^. However, safety assessment is crucial in food and health applications, considering the growing concerns regarding antibiotic resistance and virulence factors. Whole-genome sequencing (WGS) has emerged as a powerful tool to address these concerns. The utilization and advancement of genomic methodologies have made substantial contributions to enhancing our comprehension and awareness of the probiotic and biotechnological capabilities inherent in novel LAB strains^3^. European Food Safety Authority (EFSA) guidelines recommend evaluating probiotic candidates through genome sequencing, safety, pathogenicity assessment, and antibiotic resistance profiling^4^. The whole-genome analysis provides insights into antibiotic resistance, probiogenomic analysis, and bacteriocin production, contributing to food safety and alternative drug discovery^5,6^. Bacteriocins, characterized by their low cytotoxicity and safety for humans and animals, have emerged as promising molecules for various applications.

Recently, there has been a growing interest in exploring bacteriocins and their potential in human and veterinary medicine^7^. However, certain global health concerns, such as the prevalence of gastric infection of *Helicobacter pylori* and its association with gastric cancer, highlight the need for effective therapies. *H. pylori* is a pathogenic bacterium that can infect and thrive within the stomach^8^. *H. pylori* is responsible for chronic stomatitis and is currently recognized as the primary contributor to stomach cancers^9^. Therefore, eliminating *H. pylori* is vital to maintaining a healthy stomach and reducing the long-term risk of stomach cancer. Gastric *H. pylori* infection affects approximately 50% of the global population, with East Asia experiencing a high prevalence of gastric cancer^10^. Classified as a category I carcinogen, *H. pylori* can survive and colonize the gastric environment, leading to chronic inflammation and the breakdown of the protective mucosal barrier. Consequently, various gastric disorders, including gastritis, gastric ulcers, and gastric cancer, may develop^11,12^. Eradicating *H. pylori* is crucial to prevent the progression of these conditions and reduce associated risks. While triple therapy is recommended for *H. pylori* infection, rising antibiotic resistance poses significant challenges. Resistance to commonly used antibiotics, such as amoxicillin, clarithromycin, and metronidazole, has increased. Furthermore, the limitations of antibiotics, such as side effects and disruption of the intestinal flora^10^, underscore the importance of finding molecules with low antibiotic resistance and reduced side effects that can effectively combat *H. pylori*, particularly in high-risk populations. Addressing this unmet medical need requires exploring novel therapeutic options, which exhibit enhanced efficacy and improved safety profiles. Probiotics have emerged as potential alternatives to antibiotic therapy for eradicating *H. pylori*. Studies have revealed that LAB bacteriocins and antimicrobial compounds exhibit antagonistic properties against various pathogens, including *H. pylori*^13–18^. The selected suitable antimicrobials, their optimal combination, dosage, frequency, duration of treatment, and the necessity to use adjuvants are the parts of successful management of an infectious process^19^. Any disruption in the balance between beneficial and harmful microorganisms within the body can lead to an inflammatory response, resulting in epithelial dysfunction^20^. *H. pylori* can modify the host microbiome, primarily by affecting the gut microenvironment and acid–base balance. Therefore, the addition of probiotics may prove advantageous in managing inflammatory and infectious diseases^20,21^. Several studies have demonstrated that incorporating probiotics into the standard antibiotic treatment for *H. pylori* can help reduce the adverse effects of antibiotics, potentially by restoring the equilibrium of intestinal microorganisms^22^. Moreover, certain probiotics are recognized for enhancing the efficacy of *H. pylori* eradication. This is achieved through a combination of immunological and non-immunological mechanisms, such as inhibiting *H. pylori* adhesion to the mucous membrane and modifying pH levels^21^. Additionally, considering the diverse mechanisms of action of various probiotic strains, combining multiple strains of probiotics could be advantageous for enhancing the infection eradication rate^23^. Prolonged use of antibiotics can result in adverse effects, including symptoms like diarrhoea, nausea, and appetite disturbances^19^. Hence, there is an urgent need for innovative strategies to prevent and treat *H. pylori*^20^. LAB antagonistic properties hold great potential for reducing or preventing *H. pylori* infections, offering new avenues for managing this global health concern.

This study aimed to enhance our understanding of whole-genome analysis by evaluating the safety assessment, probiotic traits, and bacteriocin gene clusters of the novel *Lactococcus* sp. NH2-7C. Furthermore, the in vitro cholesterol-lowering effects, immunomodulatory activity, and probiotic properties of strain NH2-7C were investigated. Additionally, the antimicrobial substance of strain NH2-7C was partially purified, characterized, in silico investigated, and evaluated for its antimicrobial spectrum.

## Results and discussion

### Identification, characterization, and whole-genome analysis of strain NH2-7C

The strain NH2-7C, a bacteriocin-producing strain, was isolated from traditional Thai fermented pork (*Nham*) collected from Pathum Thani, Thailand. The cells of strain NH2-7C were Gram-positive, spherical or ovoid (0.64 µm × 1.23 µm) and occurring singly or in chains (Supplementary Fig. 1). Strain NH2-7C exhibited differences when compared to its closest related type strain, *L. lactis* subsp. *lactis* JCM 5805^T^ on growth in 6 and 8% NaCl and acid production from l-arabinose, d-mannitol, l-sorbose, starch, and potassium gluconate^24,25^ (Supplementary Table S1).

The 16S rRNA gene sequences showed high similarity to several *Lactococcus* species, with 99.73–99.80% to *L. lactis* subsp. *lactis* JCM 5805^T^, 99.59–99.66% to *L. lactis* subsp. *hordniae* NBRC 100931^T^, 99.18–99.12% to *L. cremoris* subsp. *tructae* L105^T^, and 99.12–99.05% to *L. cremoris* subsp. *cremoris* NCDO 607^T^. However, this study highlights the limitations of solely relying on the 16S rRNA gene for species identification, especially when distinguishing between *Lactococcus* sp. and its subspecies. In recent times, genome-based taxonomic approaches, such as ANI (< 95%)^26^, AAI (< 95–96%)^27^, and dDDH (< 70%)^28,29^, along with phylogenomic trees, have emerged as recommended thresholds for distinguishing strains belonging to distinct species. Given these insights, strain NH2-7C was selected for further investigation through whole-genome analysis in the subsequent stage of this research.

The whole-genome annotation of strain NH2-7C (accession no. CP124538-CP124541) was 2.6 Mbp, with a genomic DNA G + C content of 35.1%, N_50_ of 2,595,399, L_50_ of 1, and genome coverage of 200X. Notably, the genome size of strain NH2-7C is consistent with that of other *Lactococcus lactis* strains, including *L. lactis* UBA9852, *L. lactis* subsp. *lactis* EIP 19S, *L. lactis* subsp. *lactis* A12, and *L. lactis* subsp. *lactis* Dephy 1, with a genome size of approximately 3.0–1.71 Mbp. The phylogenomic tree revealed that strain NH2-7C was separately positioned with *L. lactis* JCM 5805^T^ and *L. lactis* subsp. *hordinae* NBRC 100931^T^ (Supplementary Fig. 2). Moreover, strain NH2-7C exhibited ANIb (94.96–86.18%), AAI (94.29–89.02%), and dDDH (63.80–31.60%) values, as shown in Supplementary Table 2, which were considerably lower than the established cut-off values for species differentiation. This study suggests that the strain represents a novel species within the genus *Lactococcus*. The Prokaryotic Genome Annotation Pipeline annotation revealed 2638 genes, comprising 2519 protein-coding genes, 38 pseudogenes, 19 rRNAs, and 62 tRNA genes. On the other hand, the Rapid Annotation of microbial genomes using Subsystems Technology (RAST) identified 2653 coding sequences (CDSs) and 81 RNA genes. The circular genome and subsystem are shown in Supplementary Figs. 3 and 4, respectively.

### Cholesterol-lowering effects

#### Bile salt hydrolase activity (BSH) and cholesterol assimilation

Strain NH2-7C displayed bile salt hydrolase (BSH) activity, evident from the formation of opaque white colonies. Moreover, the NH2-7C genome contained the *bsh* gene (Supplementary Table 3), responsible for encoding choloylglycine hydrolase. The FAO/WHO Guidelines for the Evaluation of Probiotics in Food underscore the significance of BSH activity, as it is associated with several desirable properties of probiotics, including enhanced gastrointestinal tolerance and adherence^30,31^. Additionally, BSH activity has been recognized as a pivotal factor contributing to cholesterol-lowering effects (reduced total and low-density lipoprotein (LDL) cholesterol)^32^. It is considered an additional criterion for probiotic selection and safety assurance^33^. On the contrary, Begley et al.^31^, reported that the accumulation of deconjugated bile may have negative consequences, including hindering lipid digestion, disrupting the intestine, promoting gallstone formation, and potentially leading to carcinogenic secondary bile salts. However, upon careful evaluation of the available evidence and a balanced consideration of the advantages and risks, our study proposes that bile salt deconjugation could be beneficial in the case of strain NH2-7C, as it cannot convert deconjugated bile into harmful secondary bile products. Furthermore, it is essential to note that apart from the presence of choloylglycine hydrolase, no genes associated with secondary bile salt biosynthesis were identified, which supports its overall safety profile.

Additionally, strain NH2-7C assimilated cholesterol at a rate of 49.40 ± 3.46% (Table 1). The cholesterol assimilation capacity observed in this study aligns with a comprehensive analysis conducted on the NH2-7C genome, revealing the presence of cholesterol-assimilation-associated genes (Supplementary Table 3). These genes encode membrane-related proteins capable of binding to cholesterol molecules and integrating them into the cell. This incorporation of cholesterol would limit its absorption availability, resulting in its excretion through faecal matter^34^. Thus, identifying membrane-associated genes in strain NH2-7C facilitates probiotic-driven cholesterol reduction.

**Table 1:** Cholesterol-lowering effects, gastrointestinal tolerance and adhesion ability of strains NH2-7C and *L. rhamnosus* GG.

Cholesterol-lowering effects, gastrointestinal tolerance and adhesion ability		
	*Lactococcus* sp. NH2-7C	*L. rhamnosus* GG
Cholesterol-lowering effects		
Bile salt hydrolase (BSH)	+	+
Cholesterol assimilation (%)	49.40 ± 3.46	81.40 ± 4.00
Gastrointestinal tolerance (log_10_CFU/ml)		
Simulated gastric phase (pepsin 2000 U/ml, pH 3)		
0 h	8.56 ± 0.07	9.38 ± 0.04
3 h	8.05 ± 0.14*	7.78 ± 0.18*
Simulated intestinal phase (pancreatin based on trypsin activity at 100 U/ml, pH 7)		
0 h	7.00 ± 0.15	6.68 ± 0.25
5 h	4.76 ± 0.25*	4.83 ± 0.13*
Adhesion ability (%)	68.01 ± 1.34	68.75 ± 1.24

+ , presented bile salt hydrolase activity; *, presented the significant difference of the comparison between initial time and gastric/small intestinal-emptying time (*p* < 0.05); **, presented the significant difference of the comparison between strain NH2-7C and *L. rhamnosus* GG (*p* < 0.05).

The novelty in this study includes the unique bile salt hydrolase (BSH) activity exhibited by strain NH2-7C. This activity is associated with desirable probiotic traits like improved gastrointestinal tolerance and cholesterol-lowering effects. Furthermore, the strain NH2-7C lacks genes associated with the production of harmful secondary bile salts, supporting its safety profile. Additionally, strain NH2-7C showed a significant cholesterol assimilation capacity, which is attributed to the presence of specific genes in its genome that facilitate the binding and integration of cholesterol, potentially aiding in cholesterol reduction.

#### Gastrointestinal transit tolerance

Strain NH2-7C possesses a notable strength and novelty in its exceptional resilience to gastrointestinal conditions, a crucial requirement for effective probiotics. The study evaluated its survivability following exposure to simulated gastric and small intestinal conditions, as shown in Table 1. Under simulated gastric conditions, the strain experienced only a slight decrease in viability, with the log CFU/ml shifting from 8.56 to 8.05. Subsequently, it endured small intestinal conditions and an incubation period of 5 h, resulting in a reduction in viability from 7.0 to 4.76 log CFU/ml. Of significance is that the survivability of strain NH2-7C was found to be comparable to that of the well-known probiotic *Lacticaseibacillus rhamnosus* GG. This similarity underscores the promising potential of strain NH2-7C as a probiotic candidate, given its robustness in tolerating the challenging conditions of the human gastrointestinal tract.

#### Adhesion assay

Adhesion ability is essential for probiotics to colonize and provide beneficial effects. Strain NH2-7C potentially bound to Caco-2 cells (68.01%), and its adhesion did not significantly differ from that of *L. rhamnosus* GG. The adhesion ability of strain NH2-7C is consistent with earlier studies^35–38^. Furthermore, the adhesion ability was also supported by genes encoding proteins responsible for adhesive ability, such as *ylcC, dltD, dltA, tuf, groS*, and *groL* (Supplementary Table 4). This genetic foundation, coupled with its high adhesion rate, enhances the potential of strain NH2-7C as an effective probiotic that can successfully colonize and confer beneficial effects within the host.

#### Immunomodulation effects

**Figure 1:**
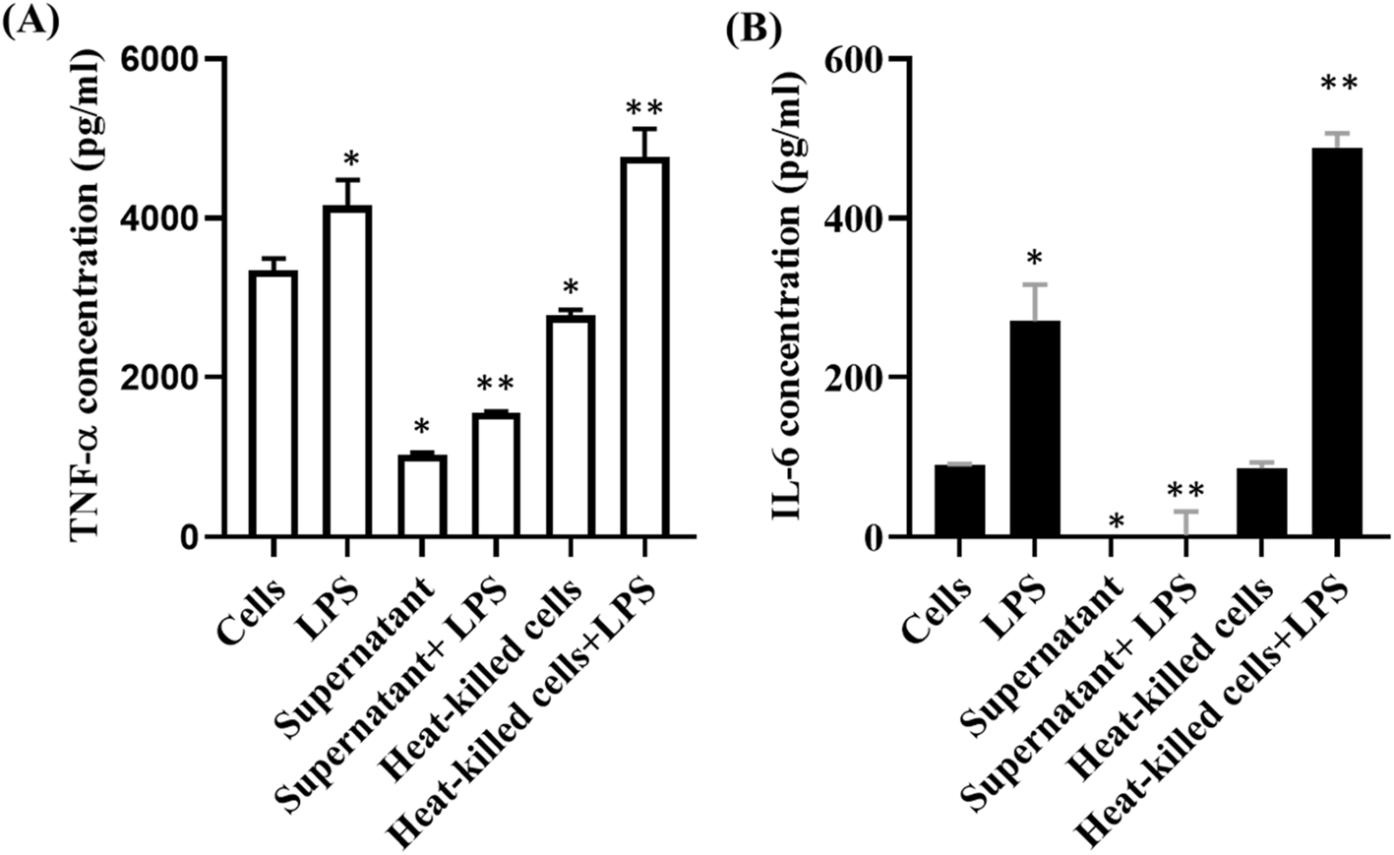
The immunomodulation effects of strain NH2-7C. The concentrations of TNF-α (**A**) and IL-6 (**B**) *p < 0.05, compared to no stimulation within each column; **p < 0.05, compared to LPS.

According to the findings, heat-killed cells of strain NH2-7C did not induce the production of TNF-α (2777.31 ± 45.29 pg/ml) and IL-6 (86.30 ± 4.86 pg/ml), respectively. However, the combination of lipopolysaccharide (LPS) and heat-killed cells resulted in a significant induction of TNF-α (4773.97 ± 221.42 pg/ml) and IL-6 (488.59 ± 11.57 pg/ml) production. While this finding indicates that some components of strain NH2-7C might enhance proinflammatory responses, it highlights the importance of closely monitoring and appropriately applying these components. The immunomodulatory effects of strain NH2-7C, as depicted in Fig. 1, are pivotal in maintaining intestinal homeostasis and overall host health. Strain NH2-7C can antagonize undesirable bacteria and promote a "physiological state of inflammation," which is crucial for specific infectious and immunological reactions^39^. Furthermore, the cell-free supernatant (CFS) exhibited anti-inflammatory activity by inhibiting the synthesis of TNF-α and IL-6. The CFS demonstrated a significant reduction in the production of these proinflammatory cytokines, similar to previous studies^40,41^. This finding highlights the potential of CFS as a promising anti-inflammatory agent.

Remarkably, even in their postbiotic form, the immunomodulatory effects remain present. This highlights the advantages of utilizing dead or inactive probiotic cells, which include a reduced risk of probiotic sepsis, antibiotic resistance, and extended shelf life^37^. However, it is important to note that careful monitoring and appropriate application of postbiotics are essential to avoid excessive inflammation. This finding suggests the potential of strain NH2-7C, with its immunomodulatory effects, to benefit immune-related disorders and potentially contribute to preventing cardiovascular diseases, which are correlated with elevated levels of IL-6 and TNF-α^42^.

##### Bacteriocin production of *Lactococcus* sp. NH2-7C

**Figure 2:**
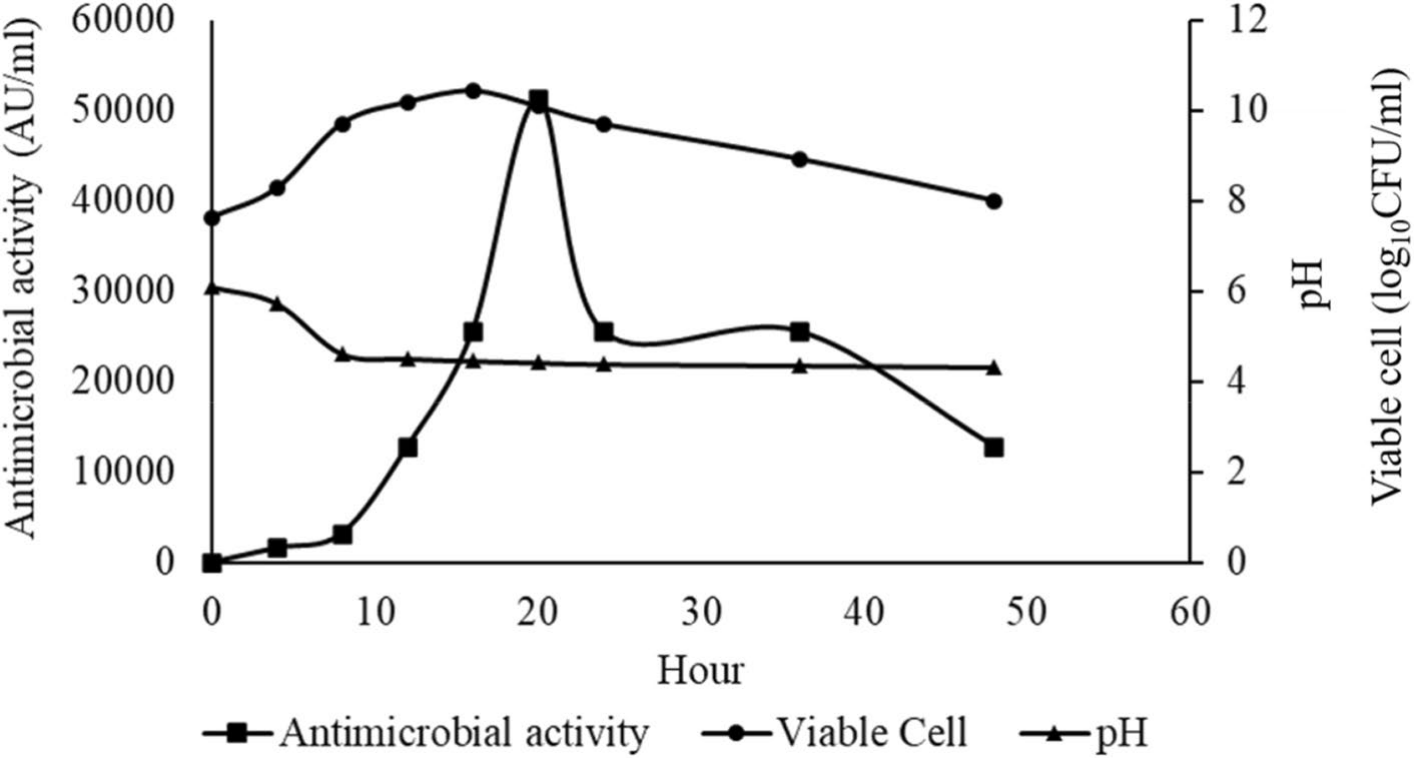
Time course of bacteriocin production by strain NH2-7C.

Figure 2 depicts the bacteriocin production of strain NH2-7C and the utilization of *Latilactobacillus sakei* JCM 1157^T^ as an indicator strain. The bacteriocin production was initiated during the early exponential growth phase at 4 h, reaching its peak activity of 51,200 AU/ml at 20 h during the late stationary phase. These findings underscore that bacteriocin production was most significant and reached its zenith during the exponential growth phase, with subsequent attenuation towards the end of the stationary phase^43^. Notably, the decline in antimicrobial activity observed after prolonged incubation could be ascribed to various factors, including proteolytic enzymes, environmental conditions, adsorption, and aggregation^44^. Additionally, as the growth progressed, a decreasing trend in pH was observed, with the initial pH of the culture medium (6.0) declining to 4.32.

##### Partial purification of antimicrobial peptide

The antimicrobial peptide derived from strain NH2-7C was partially purified using Amberlite™ XAD-16 (hydrophobic interaction chromatography) and SP-Sepharose (cation-exchange chromatography). A volume of one litre of CFS of strain NH2-7C was subjected to adsorption using Amberlite™ XAD-16, and the eluent was obtained under 20% ethanol conditions. Approximately 50% of the total bacteriocin activity in the culture supernatant was recovered using hydrophobic interaction chromatography with Amberlite™ XAD-16 resin. Subsequently, the sample underwent cation-exchange chromatography (SP-Sepharose), resulting in the recovery of bacteriocin activity within the 0.25 M NaCl fraction. After these purification steps, approximately 10% of the total activity in the culture supernatant was obtained. Table 2 presents the bacteriocin's total antimicrobial activity, yield, and purification fold.

**Table 2:** Partial purification of bacteriocin produced by *Lactococcus* sp. NH2-7C.

Fraction	Volume (ml)	Activity (AU/ml)	Total activity (AU)	Yield (%)	Total protein (mg)	Specific activity (AU/mg)
Culture supernatant	1000	51,200	5.12 × 10^7^	100	1.51 × 10^4^	3.4 × 10^3^
Amberlite™	500	51,200	2.56 × 10^7^	50	2.67 × 10^3^	9.6 × 10^3^
Sp-sepharose	50	102,400	5.12 × 10^6^	10	1.98 × 10^2^	2.6 × 10^4^

### Antimicrobial spectrum

Table 3 presents the antimicrobial spectrum of the CFS of strain NH2-7C, illustrating its inhibitory effects on a range of indicator strains. The CFS inhibited LAB indicator strains, including *Enterococcus faecalis* JCM 5803^T^, *En. faecium* JCM 5804^T^, *Lactiplantibacillus plantarum* ATCC 14917^T^, *Latilactobacillus sakei* JCM 1157^T^, *Lactococcus lactis* ATCC 19435^T^, *Leuconostoc mesenteroides* JCM 6124^T^, *Pediococcus dextrinicus* JCM 5887^T^, and *P. pentosaceus* JCM 5885, consistent with its fundamental characteristic of targeting closely related bacterial strains^45^. Additionally, the CFS of strain NH2-7C exhibited a broad antimicrobial spectrum, effectively inhibiting Gram-positive and Gram-negative bacteria. It displayed remarkable inhibition against *Helicobacter pylori* ATCC 43504^T^ (isolated from a gastric cancer patient) and two clinical *H. pylori* strains (strain 3875 and strain BK 364, isolated from patients with gastritis and gastric cancer). The worldwide prevalence of *H. pylori* infection and its consequential morbidity and mortality present challenges for conventional antibiotic-based treatments due to the emergence of antibiotic resistance in *H. pylori*. As a result, there is an increasing demand for alternative or adjunctive therapies, such as probiotics, to combat *H. pylori*, as proposed by numerous researchers. At the same time, the effects of antimicrobial peptides on *H. pylori* growth have been less extensively studied compared to other pathogens like *Listeria monocytogenes*, *Staphylococcus aureus*, *Clostridium difficile*, *Escherichia coli*, and *Salmonella* sp., further research on antimicrobial peptides with anti-*H. pylori* activity synthesized by LAB is of utmost importance. Such investigations could pave the way for developing effective strategies to tackle *H. pylori* infections and enhance treatment outcomes^46^. Additionally, *L. innocua*, *L. monocytogenes*, *S. aureus*, *St. mutans*, *St. suis* (strain NaH and P1/7 were isolated from infected patients and pigs), *Aeromonas hydrophila*, *Vibrio harveyi*, and *V. parahaemolyticus* were also inhibited, while *Candida albicans* exhibited resistance. In light of the increasing demand for chemical-free preservatives in food products, especially among health-conscious consumers, a discernible shift in the market trend is evident^44^. Strain NH2-7C's nisin A can be a potent and natural antimicrobial agent, making it a promising option for a variety of applications. These findings highlight the strain's potential for combating a range of clinical pathogens, with a particular focus on addressing *H. pylori* infections and enhancing treatment options. This multifaceted antimicrobial strength positions strain NH2-7C as a valuable candidate for addressing bacterial infections and advancing healthcare solutions.

**Table 3:** Antimicrobial spectrum of strain NH2-7C.

Indicator strain	Antimicrobial activity (AU/ml)
Gram-positive bacteria	
* Bacillus circulans* JCM 2504^T^	0
* B. subtilis* JCM 1465^T^	200
* B. cereus* ATCC 6633^T^	100
* Enterococcus faecalis* JCM 5803^T^	400
* En. faecium* JCM 5804^T^	2400
* Lactiplantibacillus plantarum* ATCC 14917^T^	400
* Latilactobacillus sakei* JCM 1157^T^	3200
* Lactococcus lactis* ATCC 19435^T^	400
* Leuconostoc mesenteroides* JCM 6124^T^	2400
* Listeria inocua* ATCC 33090^T^	100
* Li. monocytogenes* ATCC 19115	200
* Kocuria rhizophila* MIII	100
* K. luteus* NBRC 12708	150
* Pediococcus dextrinicus* JCM 5887^T^	6400
* P. pentosaceus* JCM 5885	400
* Staphylococcus aureus* ATCC 23235	0
Methilcillin-resistant *S. aureus* DMST 20635	0
* S. aureus* ATCC 25923	0
* S. aureus* ATCC 6538	200
* S. aureus* Cowan I	400
* S. aureus* DMST 6512	0
* Streptococcus agalactiae* 1611	200
* St. gordonii* DMST 35778	0
* St. iniae* SI 1810	0
* St. mutans* DMST 18777	400
* St. pyogenes* DMST 17020	0
* St. suis* NaH	400
* St. suis* P1/7	400
Gram-negative bacteria	
* A. hydrophila* B1 AhB1	400
* Campylobacter coli* NCTC 11353	0
* E. coli* ATCC 25922	0
* E. coli* O157:H7	0
* E. coli* F18	0
* E. coli* ATCC 35401	0
* E. coli* JCM 1093	0
* H. pylori* ATCC 43504^T^	6400
* H. pylori* 3875	3200
* H. pylori* BK 364	3200
* Pseudomonas aeroginosa* ATCC 27853	0
* Sal.* Typhimurium ATCC 13311^T^	0
* Vibrio algenolyticus* Va	0
* V. harveyi* AQVH 01	200
* V. parahaemolyticus* DMST 26792^T^	0
* V. parahaemolyticus *1691	800
* V. parahaemolyticus *1681	800
* V. vulnificus* 1809	0
Yeast	
* Candida albicans* ATCC 10231	0
* C. albicans* ATCC 90028	0

These findings underscore the effectiveness of the CFS of strain NH2-7C against various clinical pathogens, highlighting the desirable characteristics of strain NH2-7C. Furthermore, the study by Kim et al.^47^ supported the anti-*H. pylori* activity of partially purified bacteriocin NH2-7C, as they reported the presence of anti-*H. pylori* activity in nisin A. The research by Mota-Meira et al.^48^ revealed that *H. pylori* strains exhibited sensitivity to nisin A. These findings suggest that these compounds warrant exploration for potential development as treatments for peptic ulcers. Furthermore, these results indicate the potential utility of strain NH2-7C in combating *H. pylori*.

### Characteristics of bacteriocin NH2-7C and bacteriocin synthesis cluster gene analysis

Table 4 presents the impact of enzymes, chemicals, pH, and temperature on the antimicrobial activity of the partially purified bacteriocin from strain NH2-7C. The bacteriocin NH2-7C was subjected to enzymatic treatment. Among the various enzymes tested, proteinase-K, α-chymotrypsin, lipase, amylase, and trypsin were found to predominantly inactivate the bacteriocin NH2-7C, while pepsin had a partial inhibitory effect. This observation suggests that the inhibitory compound present in the bacteriocin NH2-7C is likely proteinaceous, consistent with previous findings by Gupta and Tiwari^49^. Furthermore, the inactivation of the partially purified bacteriocin NH2-7C by lipase and amylase aligns with the results reported in earlier studies^50,51^.

**Table 4:** Effects of enzymes, chemicals, pH and temperature on the antimicrobial activity of partially purified bacteriocin NH2-7C.

Treatment	Relative activity (%) after treatment^a^
	Partially purified bacteriocin NH2-7C
Untreated	100.00
Enzymes	
Trypsin	6.25
α-Chymotrypsin	3.13
Pepsin	50.00
Proteinase-K	1.56
Lipase	3.13
Amylase	3.13
Organic solvents	
Ethanol	100.00
Isopropanol	100.00
Acetonitrile	100.00
Chemicals	
Tween 20	100.00
Tween 80	100.00
SDS	200.00
EDTA	100.00
Triton-X-100	100.00
Urea	50.00
pH	
2	100.00
3	100.00
5	100.00
7	100.00
9	12.50
11	6.25
13	1.56
Temperatures	
100 °C, 10 min	100.00
100 °C, 20 min	100.00
100 °C, 30 min	100.00
121 °C, 15 min	50.00

^a^The activity of an untreated sample was defined as 100%.

The bacteriocin was stable under the temperature treatment at 100 °C for up to 30 min. However, when exposed to the sterilization temperature (121 °C for 15 min), the activity decreased by 50%. These findings indicate that the partially purified bacteriocin NH2-7C exhibits thermostability. Furthermore, the partially purified bacteriocin NH2-7C was not susceptible to organic solvents.

Regarding pH, the partially purified bacteriocin NH2-7C demonstrated activity over a wide pH range of 2 to 13. The activity remained stable between pH 2 and 7, while a decrease in activity was observed at pH values ranging from 9 to 13. These results highlight the pH stability of the bacteriocin within a broad range, a common characteristic of bacteriocins^52,53^. Notably, the partially purified bacteriocin NH2-7C appeared more stable under acidic conditions, which aligns with previous studies^50,51^. Nisin A is resistant to acidic conditions, making it a promising candidate as the preferred antibacterial agent against *H. pylori*. The partial inactivation of bacteriocin activity by pepsin is advantageous because it ensures safety, considering that enzymes in the human gastrointestinal tract can degrade these bacteriocins. Despite this partial enzymatic inactivation, bacteriocin NH2-7C remains effective against *H. pylori*.

Additionally, the presence of 1% (v/v) Tween 20, Tween 80, Triton X-100, and EDTA did not impact the antimicrobial activity of the bacteriocin NH2-7C. However, under 1% urea exposure, antimicrobial activity decreased, while SDS enhanced antimicrobial activity. SDS acts as a destabilizer, altering cell membrane permeability and increasing susceptibility to the bacteriocin's effects^54,55^. The sensitivity of the partially purified bacteriocin NH2-7C to chemicals showed similarities to other bacteriocins^44,56^. Nonetheless, it is essential to note that the sensitivity to surfactants and urea depends on the specific properties of the bacteriocin^44,57^.

The BAGEL v.4.0 identified a bacteriocinogenic genetic cluster in the strain NH2-7C, as depicted in Supplementary Fig. 5. The analysis revealed the presence of a gene cluster responsible for nisin A production (*nisA*, *nisB*, *nisT*, *nisC*, *nisI*, *nisP*, *nisR*, *nisK*, *nisF*, *nisE*, *nisG*) with a bit-score of 115.16 and 100% identity. Nisin has obtained FDA (Food and Drug Administration) approval for its utilization in various applications. The production of bacteriocins has gained significant recognition as a crucial attribute in selecting probiotic strains. The presence of nisin cluster genes in *Lactococcus* sp. is consistent with previous studies^58,59^.

From these findings, the bacteriocin from strain NH2-7C demonstrates robust stability against enzymes and chemicals, highlighting its potential as a reliable antimicrobial agent. It exhibits impressive thermostability, retaining activity even under sterilization conditions. The bacteriocin's wide pH tolerance and resistance to various chemicals make it versatile for different applications. Additionally, the presence of a genetic cluster for nisin A production further strengthens its potential as a valuable probiotic strain with FDA-approved attributes. These unique properties position strain NH2-7C as a promising candidate for antimicrobial applications where stability and efficacy are crucial.

### In silico evaluation of other biological activities and preliminary pharmacokinetic ADME nisin A

Remarkably, nisin A was determined to be nontoxin^60^, It was also demonstrated to have predictive antiviral activities against DENV-1 (Dengue virus), DENV-2, HIV-1 (Human immunodeficiency virus), JEV (Japanese encephalitis virus), MERS-CoV (Middle East respiratory syndrome coronavirus), SARs-COV, SARs-CoV-2 (severe acute respiratory syndrome coronavirus 2), WNV (West Nile virus), ZIKV (Zika virus), and HCV (Hepatitis C virus). However, nisin A did not exhibit predictive inhibitory effects against HSV-1 (herpes simplex virus) and VSV (vesicular stomatitis virus).

Moreover, a comprehensive evaluation of therapeutic peptides^61^ uncovered multiple beneficial properties of nisin A, including anti-angiogenic, antibacterial, anticancer, and anti-inflammatory activities. Conversely, nisin A exhibited a lack of cell-penetrating and quorum-sensing properties. Additionally, the initial pharmacokinetic evaluation of nisin A encompassing absorption, distribution, metabolism, excretion, and toxicity (ADMET) properties was conducted using the ADMETlab 2.0 (Supplementary file ADMETlab). Notably, nisin A was found to be a nonhERG (human ether-à-go-go-related gene) blocker and did not induce drug-induced liver injury (DILI). It also displayed no AMES toxicity, rat oral acute toxicity, skin sensitization, carcinogenicity, eye corrosion, irritation, or respiratory toxicity. Nevertheless, further investigations and optimal dosage determinations are imperative to evaluate and validate these promising findings comprehensively.

### Probiogenomic traits of strain NH2-7C

The genomic analysis revealed the presence of probiotic genes involved in stress response and adhesion, all of which contribute to the probiotic properties (Table 3S).

Adhesins in the probiotic cell wall are essential for adherence to the gut^62^. Adhesive genes were detected, and previous studies have suggested that sortase-dependent surface proteins play a role in mucosal adhesion processes and intestinal homeostasis^63^. Sortase class A (*srtA*) plays a vital role in the covalent attachment of LPXTG proteins to the cell wall. Among these LPXTG proteins, those containing mucus-binding domains are particularly important as they facilitate adherence to host surfaces^64^. Notably, mucus-binding proteins are crucial for establishing adherence to the intestinal mucosa^65,66^.

The *atp* operon contains essential genes, including *atpC*, *atpD*, *atpG*, *atpH*, *atpF*, *atpB*, *atpE*, and *atpA*, responsible for encoding the F_1_F_0_-ATPase. This ATPase is crucial for the survival and tolerance of the organism in acidic environments, and it plays a significant role in proton pumping, helping to maintain a neutral pH^67,68^. S-Ribosylhomocysteinase (*luxS*) plays a significant role in autoinducer-2 synthesis, which promotes stress resistance. Moreover, *luxS* gene has been linked to the ability to adhere to intestinal epithelial cells^69^. Moonlighting protein genes, including elongation factor Tu and chaperonin GroEL, are multifunctional and associated with adhesion to epithelial cells and immunomodulation^70^. Furthermore, probiotics play a crucial role in the gut by synthesizing nutrients such as fatty acids. The comprehensive genome analysis of strain NH2-7C offers valuable insights into the molecular mechanisms underlying its probiotic effects, setting the stage for future applications.

### Safety assessment of strain NH2-7C

The genomic analysis of strain NH2-7C provides compelling evidence supporting its potential as a safe strain (Table 5). When considering strains for probiotic use, it is crucial to conduct comprehensive safety assessments and evaluate their genetic makeup for any potential virulence, pathogenicity, or toxicity factors^71,72^. The strain NH2-7C’s genome presents a strong case for its probiotic potential. PathogenFinder indicated that strain NH2-7C is predicted to be a non-human pathogen, ensuring its safety for use in probiotic applications. Furthermore, genome annotation revealed the presence of genes associated with defense mechanisms, such as exopolysaccharide biosynthesis proteins and capsular polysaccharide biosynthesis proteins. Exopolysaccharides are pivotal in facilitating adhesion on biotic and abiotic surfaces, empowering bacteria to thrive in harsh environments marked by osmotic, desiccation, and oxidative stresses^73,74^. Polysaccharides also contribute to strain-specific characteristics essential for probiotic function, including stress resistance, adhesion, and immunomodulation^75^. Capsular polysaccharides have also been observed to facilitate gastrointestinal tract colonization and regulate the immune system^76^. The genome underwent analysis using the VirulenceFinder tool, which revealed the absence of recognized virulence determinants or genes linked to enterococcal surface proteins (*esp*), collagen adhesion (*ace*), serine protease (*sprE*), gelatinase (*gelE*), and cytolysins (*cylL*). This observation reinforces the Generally Recognized as Safe (GRAS) status of strain NH2-7C, as the absence of these well-established virulence genes indicates a reduced potential for harmful effects.

**Table 5:** Pathogenicity prediction, prophage region detection and antimicrobial resistance (AMR) analysis of strain NH2-7C.

Attribute/strain	Strain NH2-7C	*L. plantarum* 299v	*L. rhamnosus* GG
Probability of being a human^a^ pathogen	0.209	0.185	0.198
Input proteome coverage (%)^a^	1.74	0.48	40.5
Matched pathogenic families^a^	0	0	0
Matched not pathogenic families^a^	44	15	1147
Conclusion^a^	Non-human pathogen	Non-human pathogen	Non-human pathogen
No. of plasmid(s)^b^	2 (rep32, repUS53)	2 (rep28, rep38)	0
No. of phage region(s)^c^	9	4	5
AMR genes			
CARD^d^			
No. of perfect hits	0	0	0
No. of strict hits	2 (*tet*(s), *vanY*)	0	0
No. of loose hits	158	194	207
ResFinder^e^	*tet*(S)	No resistance	No resistance

^**a**^Data obtained from PathogenFinder; ^b^Data obtained from PlasmidFinder; ^c^Data obtained from PHASTER; and ^e^Data obtained from ResFinder.

The haemolysis III (*hlyIII*) gene was identified in the NH2-7C genome. This gene is not exclusive to this strain. However, it has been identified in various commercial probiotics, such as *L. plantarum* 299V and *L. rhamnosus* GG, both recognized as safe for consumption. On sheep-blood agar, strain NH2-7C importantly exhibited gamma haemolytic activity (non-haemolysis). In the absence of other pathogenesis genes observed in the genome, the presence of the haemolysis III gene does not pose any safety risk. Additionally, these virulence genes may confer advantages to the bacterium by enhancing its endurance, which can be beneficial in conditions that require viable bacteria, such as starters and probiotics^38^.

The potential transfer of antimicrobial resistance (AMR) genes from non-pathogenic bacteria to pathogens is a significant concern. To evaluate this risk, the study specifically investigated two types of mobile elements: plasmids and prophage regions. Strain NH2-7C possessed two plasmids similar to other *Lactococcus* and some commercially probiotic strains. Regarding determining the phage region, the NH2-7C genome contained nine prophage regions. Among these regions, two were intact, five were incomplete, and two were considered of questionable significance. Significantly, no AMR genes were identified within these prophage regions. This finding is crucial because it indicates that the strain does not possess genes associated with antibiotic resistance, reducing the risk of horizontal gene transfer. Prophage regions may also contribute to survival in harsh environments and improve adhesion ability^77–79^. Of specific significance, the genome analysis revealed the integration of four bacteriophage lysins within the prophage region. It has been previously documented that these bacteriophage lysins exhibit the capacity to impede pathogens^80^. Bacteriophage lysins exhibit the capacity to impede pathogens. Additionally, as the assigned scores indicate, the questionable significance and incomplete nature of the prophage regions further support the strain's safety. This study implies that the prophage regions do not possess characteristics associated with pathogenicity or virulence.

To determine AMR genes, the *tet*(s) gene was identified in the Resfinder 4.1 database. In contrast, the CARD database search, employing default parameters (perfect and strict hits only), detected the two genes, *tet*(*s*) and *vanY*. However, when the parameters were adjusted to include perfect, strict, and loose hits, 160 hits were obtained (2 strict hits and 158 loose hits), showing an identity range of 20–99.70% for the matching regions. The loose hits included genes associated with diverse antibiotic resistance mechanisms, encompassing antibiotic target alteration (47), antibiotic target protection (10), antibiotic efflux (95), antibiotic inactivation (4), and antibiotic target replacement (4). The Resfinder and CARD databases primarily concentrate on antimicrobial resistance genes in pathogenic bacteria, often overlooking those present in non-pathogenic bacteria.

In contrast, our exploration of the KEGG database revealed six AMR-related genes in the NH2-7C genome (Table 6). Among these genes, the *vanY* gene was linked to vancomycin resistance, a trait inherent in LAB strains that synthesize D-lactate-ended peptidoglycan precursors instead of D-alanine at the C-terminus^81,82^. The *vanY* gene encodes for D-Ala-D-Ala carboxypeptidase. Additionally, cationic antimicrobial peptide resistance genes were found in the genome. However, the presence of AMR genes in the genome does not necessarily guarantee resistance, as proposed by Chokesajjawatee et al.^72^. The authors further elucidated that gene expression and substrate specificity could significantly influence the actual resistance phenotype. Importantly, all the AMR-related genes identified in *Lactococcus* sp. NH2-7C were also found in other probiotics, such as strains 299V, JDM1, ST-III, and WCFS1^72^, indicating their widespread presence within the probiotics. Hence, it has been observed in certain previous studies that, despite AMR genes, LAB strains may still exhibit phenotypic susceptibility to common antibiotics, underscoring that the correlation between phenotype and genotype may not always be absolute. Therefore, more extensive studies are necessary to ascertain whether these potential AMR genes encode active proteins or serve distinct functional roles.

**Table 6:** AMR (antimicrobial resistance) genes identified in the *Lactococcus* sp. NH2-7C genome.

Resistance	Description	KEGG_ID	Gene name
Vancomycin resistance	d-Ala-d-Lac type [MD:M00651]	K07260	*vanY*; zinc d-Ala-d-Ala carboxypeptidase [EC:3.4.17.14]
Cationic antimicrobial peptide (CAMP) resistance	dltABCD operon [MD:M00725]	K03367	*dltA*; d-alanine-poly(phosphoribitol) ligase subunit 1 [EC:6.1.1.13]
		K03739	*dltB*; membrane protein involved in d-alanine export
		K14188	*dltC*; d-alanine-poly(phosphoribitol) ligase subunit 2 [EC:6.1.1.13]
		K03740	*dltD*; d-alanine transfer protein
Multidrug resistance	Efflux pump AbcA [MD:M00700]	K18104	*abcA, bmrA;* ATP-binding cassette, subfamily B, bacterial AbcA/BmrA [EC:7.6.2.2]

All data were obtained from KEGG database.

Evaluating biogenic amine (BA) productions also hold significant implications. Following the guidelines issued by the European Food Safety Authority (EFSA), it is recommended to adopt BA-nonproducing strain as a precautionary measure to mitigate the potential risks associated with BAs^83^. To ascertain the BA-nonproducing nature and safety of a given strain in this context, it is possible to investigate the absence of genes responsible for BA synthesis. Should these genes be lacking, the strain can be deemed BA-nonproducing and regarded as safe. According to the KEGG annotation, strain NH2-7C lacks genes related to biogenic amine (BA) production, including tryptamine, tyramine, histamine, ornithine, spermine, spermidine, putrescine, and cadaverine, within its genome. Consequently, strain NH2-7C can be categorized as a BA nonproducer, posing no safety concerns.

Additionally, the genome of strain NH2-7C has been annotated with the l-lactate dehydrogenase (L-LDH) gene. The absence of lactate racemase and D-lactate dehydrogenase (D-LDH) proves advantageous, rendering it suitable for probiotic and starter applications. d-lactate production can potentially lead to d-lactate acidosis^84^.

From the comprehensive investigation, strain NH2-7C presents unique and distinctive features for probiotic applications. Its genome underscores its safety, as it lacks virulence factors and antimicrobial resistance genes. It exhibits stress resistance, pH stability, and does not produce biogenic amines. Moreover, its genetic makeup is well-suited for probiotic and starter applications. These characteristics position NH2-7C as a promising and original probiotic candidate, warranting further research to fully explore its potential.

### Carbohydrate-active enzyme analysis

Strain NH2-7C demonstrates a notable abundance of genes associated with carbohydrate metabolism, indicating its high adaptability and potential interactions with the human host.

Strain NH2-7C comprised 88 carbohydrate-active enzyme genes (Supplementary file CAZY). Among these, there are 28 glycosyltransferase (GT) genes, 44 glycoside hydrolase (GH) genes, eight carbohydrate esterase (CE) genes, seven carbohydrate-binding molecules (CBMs), and one auxiliary activity (AA) gene. These findings highlight NH2-7C's capacity to utilize many mono- and polysaccharides as energy sources while synthesizing complex compounds. The GH enzyme families within strain NH2-7C reveal key oligosaccharide-degrading enzymes, such as GH13 and GH32. Oligosaccharides, known for their prebiotic properties, are vital in maintaining a healthy gut^38^. Furthermore, GH families are essential for the hydrolysis and synthesis of oligosaccharides, which can serve as selective prebiotics^70^. Furthermore, glycosyltransferases (GTs) allow it to catalyse the transfer of sugars from activated donor molecules to specific acceptors. This ability to utilize various carbohydrates is essential for constructing surface structures recognized by the host immune system, underscoring its significance in the overall process^38^. Hence, strain NH2-7C possesses these genetic attributes, enabling carbohydrate utilization and indicating its potential as a probiotic, with implications for immunomodulation and the prevention of pathogen-related complications.

## Conclusion

In conclusion, this study focused on the novel species of *Lactococcus* sp. NH2-7C, which was isolated from fermented pork, and examined its bacteriocin and probiotic properties. Comparative genotypic analysis indicates that NH2-7C represents a novel species within the genus *Lactococcus*, and in silico assessments confirm its non-pathogenic nature and the absence of virulence-associated genes. Moreover, the presence of the *nisA* gene responsible for nisin A production suggests its potential as a non-toxic, anti-*H. pylori* compound. Probiotic assessments have shown that strain NH2-7C possesses bile salt hydrolase and cholesterol assimilation activities, and the ability to modulate cytokine secretion and adhere to Caco-2 cells, affirming its safety and probiotic potential. Additionally, its bacteriocin production further supports its suitability as a functional probiotic strain with therapeutic potential. However, the study underscores the need for further in vivo investigations to ensure the safety of *Lactococcus* sp. NH2-7C and explore its potential applications as a probiotic agent. These future studies will be crucial in fully realizing the probiotic and therapeutic capabilities of this novel *Lactococcus* sp. NH2-7C, particularly in combatting gastric diseases associated with *H. pylori*.

## Experimental procedures

### Characterization of strain NH2-7C

Strain NH2-7C was characterized following the methods described by Kingkaew et al.^38^, which included phenotypic and chemotaxonomic analyses. The extraction of genomic DNA was carried out using the Wizard Genomic DNA Purification kit (Promega Corporation, USA) following the manufacturer’s instructions. A combination of hybrid technologies was utilized for whole-genome sequencing and obtaining comprehensive genomic information. Specifically, Oxford Nanopore Technologies (ONT) was employed with the Rapid sequencing kit and MinIONTM device (Oxford Nanopore Technologies, UK), offering a long-read sequencing approach. In contrast, the Illumina platform with the NextSeq^®^ 500 high output kit v2 (300 cycles) (Illumina, Inc., USA) enhanced sequencing accuracy. Removing adaptors and low-quality reads was carried out to ensure high-quality data using Trim Galore (Galaxy Version 0.6.3). Subsequently, the filtered reads were subjected to genome assembly using the Unicycler program (Galaxy Version 0.4.8.0). The sequence similarity between strain NH2-7C and reference strains was assessed using the EzBiocloud tool^85^. The calculation of average nucleotide identity (ANI) and digital DNA-DNA hybridization (dDDH) values was conducted by JSpeciesWS^26,86^ and the Genome-to-Genome Distance Calculator (GGDC 2.1) with the BLAST+ method and formula 2^87^. The Kostats lab^88^ performed the average amino acid identity (AAI). A circular genomic map and phylogenomic tree were constructed using Proksee^89^ and TYGS^90^, respectively.

### Functional genome analysis of strain NH2-7C

The genome annotation process involved the utilization of several tools and databases. Annotations were performed using the DFAST server^91^, Rapid Annotation Server Technology (RAST)^92^, PATRIC^93^, and the NCBI Prokaryotic Genome Annotation Pipeline (PGAP)^94^. Antibiotic resistance genes were identified through the Comprehensive Antibiotic Resistance Database (CARD)^95^ and ResFinder^96^ 4.1. The PathogenFinder^97^ was utilized to predict pathogenicity, and plasmids were detected using PlasmidFinder^98^. Identification and annotation of putative prophage sequences were carried out using the PHAge Search Tool Enhanced Release (PHASTER)^77^. Carbohydrate-active enzymes were identified using the dbCAN meta server (https://bcb.unl.edu/dbCAN2/) with HMMER, and the data were based on the family classification from the CAZy database (http://www.cazy.org/)^99,100^. For the bacteriocin cluster analysis, BAGEL4 (BActeriocin GEnome mining tool) was used^101^. Biological activities, toxicity, and preliminary pharmacokinetic ADME of nisin A were assessed through PreTP-Stack^60,61^ and ADMETlab 2.0^102^. The Kyoto Encyclopedia of Genes and Genomes (KEGG) database was utilized to explore pathways and genes (https://www.kegg.jp)^103^.

### Determination of cholesterol-lowering effects

#### Bile salt hydrolase (BSH) activity

The BSH activity was determined according to the method described by Kingkaew et al.^37^. To perform the assay, 20 µl of the overnight culture broth was placed as a spot-on MRS agar supplemented with 0.5% (w/v) taurodeoxycholic acid sodium salt (TDCA) (Sigma, India) and 0.037% (w/v) calcium chloride. The plates were then incubated anaerobically at 37 °C for 72 h. The presence of halos around the colonies or the appearance of opaque white colonies indicated the presence of bile salt hydrolase activity. Nonmodified MRS agar was used as the control.

#### Cholesterol assimilation ability

The ability of strain NH2-7C to assimilate cholesterol was assessed in MRS broth supplemented with cholesterol-polyethylene glycol (PEG) 600 (Sigma, India) at a final concentration of 100 µg/ml. A 1% cell suspension of NH2-7C was inoculated into the MRS-cholesterol-PEG 600 medium and incubated anaerobically at 37 ℃ for 24 h. After the incubation period, the cholesterol in the MRS broth was extracted using a method described by Tomaro-Duchesneau et al.^104^. To quantify the residual cholesterol, a modified technique from Rudel and Morris^105^ was employed. The cholesterol concentration was determined by reading the values from a standard curve prepared using a cholesterol stock solution. The ability of the probiotics to assimilate cholesterol in MRS was reported as the percentage of cholesterol removed at each incubation interval.

$${\text{Cholesterol}}\;{\text{assimilated}}\left( {\upmu {\text{g/ml}}} \right) = \left[ {{\text{Cholesterol}}\left( {\upmu {\text{g/ml}}} \right)} \right]_{{0\;{\text{h}}}} - \left[ {{\text{Cholesterol}}\left( {\upmu {\text{g/ml}}} \right)} \right]_{{24\;{\text{h}}}}$$

$${\text{\% Cholesterol}}\;{\text{assimilated}} = \left[ {\frac{{{\text{Cholesterol}}\;{\text{assimilated}}\left( {\upmu {\text{g/ml}}} \right)}}{{{\text{Cholesterol}}\left( {\upmu {\text{g/ml}}} \right)_{{0\;{\text{h}}}} }}} \right] \times 100$$

### Evaluation of probiotic properties

#### LAB cell suspension preparation

For the assessment of probiotic properties, strain NH2-7C was cultured twice in MRS broth at 30 °C for 24 h. After incubation, the cells were harvested by centrifugation at 14,000 rpm for 10 min at 4 °C. Subsequently, the cells were washed twice with phosphate-buffered saline (PBS; 0.1 M, pH 7.2, containing 0.85% (w/v) NaCl) and then resuspended in phosphate buffer (0.1 M, pH 7.0) to achieve a cell suspension with an optical density OD_600_ of 1 and a concentration of 10^9^ CFU/ml.

#### Viability during gastrointestinal (GIT) transit

Viability characteristics during gastrointestinal tract transit were assessed in vitro using a simulated gastric and intestinal fluid model, following a modified procedure based on Minekus et al.^106^. The cell suspension was mixed with simulated gastric fluid (SGF) containing pepsin (2000 U/ml) and incubated anaerobically at pH 3 and 37 °C for 3 h. Afterward, the gastric chyme was combined with simulated small intestinal fluid (SIF) containing pancreatin (with trypsin activity at 100 U/ml) and bile (10 mM) and incubated anaerobically at pH 7 and 37 °C for an additional 5 h. Samples were collected at specific time points, including 0 h (initial time), 3 h (gastric-emptying time), 0 h (initial time of intestinal transit), and 5 h (small intestinal-emptying time). A serial tenfold dilution and the spot plate method performed viable LAB quantification. As a control, *L. rhamnosus GG* was employed. The viable cells were informed as logarithms of colony-forming units (log CFU/ml).

### Adhesion assay

The strain NH2-7C was selected to assess its adhesion ability, as described by Kingkaew et al.^36^. Caco-2 cells were cultured in Dulbecco’s Modified Eagle Medium (DMEM) containing 10% (v/v) fetal bovine serum (FBS) and 1% (v/v) penicillin–streptomycin (PS) at 37 °C under a humidified condition of 5% CO_2_. Caco-2 cells were seeded at a 5 × 10^5^ cells/ml in 24-well tissue culture plates. The plates were then incubated at 37 °C in 5% CO_2_ for 48 h.

Next, the Caco-2 cells were gently cleansed twice with PBS, and the bacterial cells were harvested by centrifugation at 14,000 rpm for 10 min at 4 °C. The bacterial cells were resuspended in DMEM supplemented with no antibiotics and then added to the wells, where they were incubated for 90 min at 37 °C in 5% CO_2_. After the incubation, the Caco-2 cells were cleansed three times with PBS to remove any unbound bacterial cells. Subsequently, the cells were lysed using a 0.05% Triton-X100 solution, and the adherent bacteria was determined using the spot plate technique on MRS agar. The plates were then incubated at 37 °C for 48 h. As a control, *L. rhamnosus* GG was employed. The adherent ability was calculated using the method previously described by Alp and KuleaŞan^107^:

$${\text{Adhesion percentage (\% ) = }}\frac{{{\text{N}}_{{\text{t}}} }}{{{\text{N}}_{{0}} }} \times {100}$$

where N_t_ = the log CFU of adherent bacterial cells to the Caco-2 cells, N_0_ = the log CFU of inoculated bacterial cells.

### Haemolytic activity

Haemolytic activity is an important characteristic in determining the virulence of probiotic bacteria. To assess this activity, strain NH2-7C was streaked on sheep blood agar and incubated at 37 °C for 48 h. The resulting zones around the colonies were observed and categorized as β-haemolysis (clear zone), α-haemolysis (green-hued zone), or γ-haemolysis (no zone)^108^.

### Immunomodulatory effects

The immunomodulatory effects of strain NH2-7C were evaluated as described by Sitdhipol et al.^109^. To prepare heat-killed cells, strain NH2-7C was cultured twice in MRS broth at 30 °C for 24 h. The bacterial cells were then collected by centrifugation at 10,000 rpm for 10 min and subjected to heat treatment at 85 °C for 10 min. The resulting lysate was filtered using a 0.22 µm filter and subsequently lyophilized before conducting the test. Moreover, the supernatant of strain NH2-7C was collected after centrifugation at 10,000 rpm for 10 min, filtered through a 0.22 µm filter, and lyophilized prior to the test.

### THP-1 cell culture

THP-1 cells were cultured in RPMI-1640 medium. The cells were incubated at 37 °C in 5% CO_2_ for 48 h. A suspension of 500 µl of THP-1 cells was seeded in each well of a 24-well plate at a density of 1 × 10^5^ cells/ml with phorbol 12-myristate 13-acetate (PMA) at a concentration of 60 ng/ml. The cells were then incubated at 37 °C in 5% CO_2_ for 48 h to induce differentiation. After differentiation, the medium was replaced without PMA and incubated for 24 h. Heat-killed cells or CFS (500 µl) were added to the differentiated THP-1 cells with and without lipopolysaccharide (LPS, 1 µg/ml), followed by incubation for 1 day. The supernatants were collected, and quantified the level of TNF-α and IL-6. The production of TNF-α and IL-6 was quantified using the sandwich TNF-α DuoSet and human IL-6 DuoSet ELISA kits (R&D systems, USA), respectively. The ELISA technique followed the manufacturer's instructions (R&D Systems, USA).

### Time course of bacteriocin production and partial purification

#### Time course of bacteriocin production

Time course of bacteriocin production of strain NH2-7C was conducted as described by Woraprayote et al.^44^ with modification. An overnight culture of strain NH2-7C was inoculated into 200 ml MRS broth and incubated at 30 °C. Samples were gathered and documented at 4-h intervals for 48 h. The growth of the bacterium (log CFU/ml) was measured using a plate count method on MRS agar, while the pH changes were monitored using a pH meter. The antimicrobial activity was assessed using the spot-on-lawn assay^44^ with *Latilactobacillus sakei* JCM 1157^ T^ as the indicator strain, and it was expressed in arbitrary activity units (AU/ml). Antimicrobial activity of the CFS was quantified in activity units (AU) per millilitre. The AU value represented the reciprocal of the maximal dilution at which growth limitation was still detectable.

The formula used to calculate the titre is as follows

$$\left( {\text{AU/ml}} \right) \, = \, \left( {{2}^{{\text{N}}} } \right) \, \times { 1}00$$

where AU: arbitrary unit, N: the maximal two-fold serial dilution showing a transparent limitation zone of the indicator strain.

### Partial purification

Partial purification of the antimicrobial compound was performed according to the method described by Woraprayote et al.^44^. The procedure commenced by inoculating an overnight culture of strain NH2-7C into 1 L of MRS broth, followed by incubation at 30 °C for 20 h in aerobic conditions. Subsequently, the CFS was obtained by centrifuging the culture at 8000×*g* for 15 min at 4 °C. The antimicrobial peptide was extracted using a series of techniques, starting with hydrophobic interaction chromatography using Amberlite™ XAD-16 polymeric resin, followed by fast flow cation-exchange chromatography utilizing SP-Sepharose resin. The chromatographic steps involved a stepwise gradient from 0.25 to 1.0 M NaCl in 20 mM sodium phosphate buffer at pH 5.7. All fractions collected from the chromatography were evaluated for antimicrobial activity using the spot-on-lawn technique. The protein concentration in the fractions was determined using the Lowry^110^ method with bovine serum albumin (BSA) as the standard.

### Antimicrobial spectrum

The antimicrobial spectrum was determined using the CFS at pH 7.0 through the spot-on-lawn technique^44^. Indicator strains used for assessing the antimicrobial spectrum (Table 3) were cultured for 18 h under optimal conditions as the respective culture collections. Prior to use, all indicator strains were kept at − 80 °C with 15% glycerol and cultured in their respective media following the recommended conditions for 18 h.

### Characterization of partially purified bacteriocin NH2-7C

The impact of enzymes, chemicals, pH, and temperature on the activity of the partially purified bacteriocin from strain NH2-7 was also assessed. First, the sensitivity of the bacteriocin to various enzymes was assessed following the method described by Woraprayote et al.^44^. The partially purified bacteriocin was incubated with various enzymes at a final concentration of 1.0 mg/ml in the appropriate buffers. The enzymes included amylase (pH 7.5), pepsin (pH 3.0), α-chymotrypsin (pH 7.5), trypsin (pH 7.5), lipase (pH 7.5), and proteinase K (pH 7.5). The incubation was carried out at 37 °C for 5 h. A control sample was prepared by excluding the enzyme treatment from the partially purified bacteriocin.

Moreover, the impact of chemicals, such as organic solvents and surfactants, on the antimicrobial activity of the bacteriocin was examined. The bacteriocin was combined with different organic solvents, including isopropanol, acetonitrile, ethanol, and acetone, at a 1:1 ratio. Untreated partially purified bacteriocin and organic solvent mixed with an equal volume of sterile distilled water served as control. After thorough mixing, all samples were incubated at 30 °C for 5 h before the antimicrobial test was conducted.

To examine the heat and pH stability of the partially purified bacteriocin NH2-7C, it was adjusted to pH values ranging from 2.0 to 13.0 using 1 M HCl or 1 M NaOH. The preparations were then incubated at 100 °C for varying durations of 10, 20, and 30 min and at 121 °C for 15 min. Following the incubation period, the sample was neutralized to pH 6.5. Moreover, the antimicrobial activity of the partially purified bacteriocin without heat treatment was set as the reference value of 100%.

The remaining antimicrobial activities of the treated and control samples were evaluated using the critical dilution spot-on-lawn method, utilizing *L. sakei* JCM 1157^ T^ as the indicator strain.

### Statistical analysis

The experiments were conducted in triplicate, and the results are presented as the mean ± standard deviation (SD). Statistical analysis was performed using SPSS 22.0 software, applying ANOVA. Mean values were compared using Duncan's Multiple Range Test (DMRT) at a significance level of *p* < 0.05.

### Supplementary Information

Supplementary Information 1.

Supplementary Information 2.

Supplementary Information 3.

## Data Availability

This whole genome project has been deposited in the NCBI BioProject, BioSample, and GenBank under the following respective accession numbers: PRJNA764020, SAMN34262999, and CP124538–CP124541.
