# Supplementary material for: Functional genome analysis and anti-Helicobacter pylori activity of a novel bacteriocinogenic Lactococcus sp. NH2-7C from Thai fermented pork (Nham)
Source: Sci Rep. 2023 Nov 21;13:20362. doi: 10.1038/s41598-023-47687-4 (PMC10663479; doi:10.1038/s41598-023-47687-4)
Supplement: Supplementary file 1 — Supplementary Information 1. [file 41598_2023_47687_MOESM1_ESM.pdf]

ADMETlab 2.0 Home Services Resources Explanation Publications Contact

Home / ADMET Evaluation / Evaluation Results

CC[C@H](C)[C@H](N)C(=O)N\C(=C/C)\C(=O)N[C@@H]1CSC[C@H](NC(=O)[C@H](CC(C)C)NC(=O)C(=C)NC(=O)[C@@H](NC1=O)[C@@H](C)CC)C(=O)N[C@@H]2[C@H](C)SC[C@H](NC(=O)CNC(=O)[C@H]3CCCN3C2=O)C(=O)N[C@@H](CCCCN)C(=O)N[C@@H]4[C@H](C)SC[C@H](NC(=O)CNC(=O)[C@H](CNSC)NC(=O)[C@H](CC(C)C)NC(=O)[C@H](C)NC(=O)CNC4=O)C(=O)N[C@@H](CC(=O)N)C(=O)C[C@@H](CCSC)C(=O)N[C@@H](CCCCN)C(=O)N[C@@H]5[C@H](C)SC[C@@H]6NC(=O)[C@H](NC(=O)[C@H](C)NC5=O)[C@H](C)SC[C@H](NC(=O)[C@H](Cc7cnc[nH]7)NC6=O)C(=O)N[C@@H](CO)C(=O)NC([C@@H](C)CC)C(=O)N[C@@H](Cc8cnc[nH]8)C(=O)N[C@@H](C(C)C)C(=O)NC(=C)C(=O)N[C@@H](CCCCN)C(=O)O

Download as CSV

Download as PDF

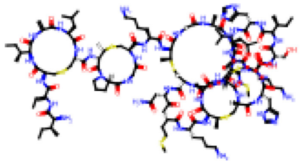

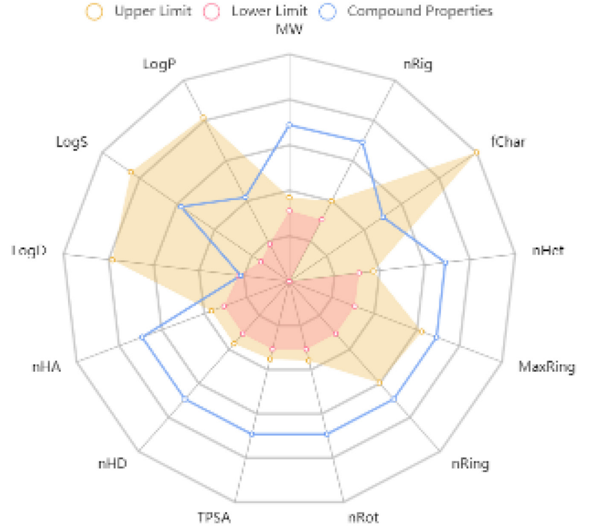

Physicochemical Property

Molecular Weight (MW)	3351.550
Volume	3219.365
Density	1.041
nHA	79
nHD	46
nRot	82
nRing	8
MaxRing	22
nHet	86
fChar	0
nRig	126
Flexibility	0.651
Stereo Centers	35
TPSA	1213.570
logS	-1.239
logP	1.109
logD	0.963

Medicinal Chemistry

QED	0.017
SAscore	9.854
Fsp^3	0.671
MCE-18	338.305
NPscore	0.448
Lipinski Rule	Rejected
Pfizer Rule	Accepted
GSK Rule	Rejected
Golden Triangle	Rejected
PAINS	0 alert(s)
ALARM NMR Rule	3 alert(s) DETAIL
BMS Rule	3 alert(s) DETAIL
Chelator Rule	0 alert(s)

Absorption

Caco-2 Permeability	-7.110
MDCK Permeability	8.4e-07
Pgp-inhibitor	---
Pgp-substrate	+++
HIA	+++
$F_{20\%}$	+++
$F_{30\%}$	+++

Distribution

PPB	70.306%
VD	0.075
BBB Penetration	---
Fu	13.729%

Metabolism

CYP1A2 inhibitor	---
CYP1A2 substrate	---
CYP2C19 inhibitor	---
CYP2C19 substrate	---
CYP2C9 inhibitor	---
CYP2C9 substrate	---
CYP2D6 inhibitor	---
CYP2D6 substrate	---
CYP3A4 inhibitor	---
CYP3A4 substrate	---

Excretion

CL	-1.452
$T_{1/2}$	0.883

Toxicity

hERG Blockers	---
H-HT	+++
DILI	---
AMES Toxicity	---
Rat Oral Acute Toxicity	--
FDAMDD	---
Skin Sensitization	--
Carcinogencity	---
Eye Corrosion	---
Eye Irritation	---
Respiratory Toxicity	---
**Environmental Toxicity**	
Bioconcentration Factors	-0.004
IGC_{50}	4.514
$LC_{50}FM$	4.155
$LC_{50}DM$	6.274
**Tox21 Pathway**	
NR-AR	---
NR-AR-LBD	---
NR-AhR	---
NR-Aromatase	---
NR-ER	--
NR-ER-LBD	+++
NR-PPAR-gamma	---
SR-ARE	-
SR-ATAD5	++
SR-HSE	---
SR-MMP	-
SR-p53	--
**Toxicophore Rules**	
Acute Toxicity Rule	0 alert(s)
Genotoxic Carcinogenicity Rule	0 alert(s)
NonGenotoxic Carcinogenicity Rule	0 alert(s)
Skin Sensitization Rule	5 alert(s) DETAIL
Aquatic Toxicity Rule	3 alert(s) DETAIL
NonBiodegradable Rule	1 alert(s) DETAIL
SureChEMBL Rule	1 alert(s) DETAIL
FAF-Drugs4 Rule	8 alert(s) DETAIL

Tip: For the classification endpoints , the prediction probability values are transformed into six symbols: 0-0.1(---), 0.1-0.3(--), 0.3-0.5(-), 0.5-0.7(+), 0.7-0.9(++), and 0.9-1.0(+++).

https://admetmesh.scbdd.com/ (Accessed on: 22 June 2023)
