# Supplementary material for: Functional genome analysis and anti-Helicobacter pylori activity of a novel bacteriocinogenic Lactococcus sp. NH2-7C from Thai fermented pork (Nham)
Source: Sci Rep. 2023 Nov 21;13:20362. doi: 10.1038/s41598-023-47687-4 (PMC10663479; doi:10.1038/s41598-023-47687-4)
Supplement: Supplementary file 2 — Supplementary Information 2. [file 41598_2023_47687_MOESM2_ESM.docx]

**Functional genome analysis and anti-*Helicobacter pylori* activity of a novel bacteriocinogenic *Lactococcus* sp. NH2-7C from Thai fermented pork (Nham)**

**Engkarat Kingkaew^1,6^, Weerapong Woraprayote^2^, Auttaporn Booncharoen^2^, Kanidta Niwasabutra^3^, Thitiphorn Janyaphisan^2^, Ratha-Korn Vilaichone^4^,** **Yoshio Yamaoka^5^ Wonnop Visessanguan^2*^, Somboon Tanasupawat^6**^**

^1^Department of Biology, School of Science, King Mongkut’s Institute of Technology Ladkrabang, Bangkok, 10520, Thailand

^2^National Center for Genetic Engineering and Biotechnology (BIOTEC), National Science and Technology Development Agency (NSTDA), Pathum Thani, 12120, Thailand

^3^Thailand Institute of Scientific and Technological Research (TISTR) Biodiversity Research Centre, Pathum Thani 12120, Thailand

^4^GI Unit, Department of Medicine, and Center of Excellence in Digestive Diseases, Thammasat University, Thailand Science Research and Innovation Fundamental Fund, Bualuang ASEAN Chair Professorship at Thammasat University, Pathum Thani 12120, Thailand

^5^Department of Environmental and Preventive Medicine, Faculty of Medicine Oita University, Yufu, Oita, Japan

^6^Department of Biochemistry and Microbiology, Faculty of Pharmaceutical Sciences, Chulalongkorn University, Bangkok 10330, Thailand

***Corresponding author:** Wonnop Visessanguan, wonnop@biotec.or.th

****Corresponding author:** Somboon Tanasupawat, Somboon.T@chula.ac.th

**Supplementary Tables**

Table S1 Phenotypic characteristics of strain NH2-7C and JCM 5805^T^.

| **Characteristics** | **NH2-7C** | **JCM 5805^T^** |
| --- | --- | --- |
| Cell shape | Cocci | Cocci |
| Catalase | - | - |
| Gas from glucose | - | - |
| Growth in 6% NaCl | + | - |
| Growth in 8% NaCl | + | - |
| Growth at pH 3 | + | + |
| pH 9 | - | - |
| Growth at 15 ºC | + | + |
| 45 ºC | - | - |
| Arginine hydrolysis | + | + |
| Nitrate reduction | - | ND |
| **Acid from:** |  |  |
| Amidon (starch; AMD) | W | - |
| Amygdalin (AMY) | + | + |
| Arbutin (ARB) | + | + |
| D-Adonitol (ADO) | - | - |
| D-Arabinose (D-ARA) | - | - |
| D-Arabitol (D-ARL) | - | - |
| D-Cellobiose (CEL) | + | + |
| D-Fructose (FRU) | + | + |
| D-Fucose (D-FUC) | - | - |
| D-Galactose (GAL) | + | + |
| D-Glucose (GLU) | + | + |
| D-Lactose (bovine origin; LAC) | + | + |
| D-Lyxose (LYX) | - | - |
| D-Maltose (MAL) | + | + |
| D-Mannitol (MAN) | + | - |
| D-Mannose (MNE) | + | + |
| D-Melezitose (MLZ) | - | - |
| D-Melibiose (MEL) | - | - |
| D-Raffinose (RAF) | - | - |
| D-Ribose (RIB) | + | + |
| D-Saccharose (sucrose; SAC) | + | - |
| D-Sorbitol (SOR) | - | - |
| D-Tagatose (TAG) | - | - |
| D-Trehalose (TRE) | + | + |
| D-Turanose (TUR) | - | - |
| Dulcitol (DUL) | - | - |
| D-Xylose (D-XYL) | + | + |
| Erythritol (ERY) | - | - |
| Esculin ferric citrate (ESC) | + | + |
| Gentiobiose (GEN) | W | W |
| Glycerol (GLY) | - | - |
| Glycogen (GLYG) | - | - |
| Inositol (INO) | - | - |
| Inulin (INU) | - | - |
| L-Arabinose (L-ARA) | + | - |
| L-Arabitol (L-ARL) | - | - |
| L-Fucose (L-FUC) | - | - |
| L-Rhamnose (RHA) | - | - |
| L-Sorbose (SBE) | + | - |
| L-Xylose (L-XYL) | - | - |
| Methyl-alpha-D-glucopyranoside (MDG) | - | - |
| Methyl-alpha-D-mannopyranoside (MDM) | - | - |
| Methyl-beta-D-xylopyranoside (MDX) | - | - |
| N-Acetylglucosamine (NAG) | + | + |
| Potassium 2-ketogluconate (2KG) | - | - |
| Potassium 5-ketogluconate (5KG) | - | - |
| Potassium gluconate (GNT) | W | - |
| Salicin (SAL) | + | + |
| Xylitol (XLT) | - | - |
| Isomer of lactic acid | L | L |
| *meso-*DAP | - | - |

+, positive reaction; w, weakly positive reaction; -, negative reaction

*Data from Schleifer et al. (1985), and Garvie & Farrow (1982).

**Table 2S** ANIb, AAI and the digital DNA-DNA hybridization (dDDH) values between the genomes of the putatively new strain NH2-7C; *Lactococcus lactis* subsp. *lactis* JCM 5805^T^; *L. lactis* subsp. *hordniae* NBRC 100931^T^; *L. cremoris subsp. cremoris* HP^T^ and *L. cremoris subsp. tructae* DSM 21502^T^

Genomic data: 1, strain NH2-7C (CP124538- CP124541); 2, *Lactococcus lactis* subsp. *lactis* JCM 5805^T^ (BBSI00000000); 3, *L. lactis* subsp. *hordniae* NBRC 100931^T^ (BCVL00000000); 4, *L.* *cremoris* subsp. *cremoris* HP^T^ (LIYE00000000); and 5*,* *L. cremoris* subsp. *tructae* DSM 21502^T^ (JXKC00000000)

| **Query genome** | **Reference genome** | **ANIb** | **AAI** | **% dDDH (Formular 2*)** | **Model C.I. (%)** | **Distance** | **Prob. DDH >= 70%** | **G+C difference** |
| --- | --- | --- | --- | --- | --- | --- | --- | --- |
| 1 | 2 | 94.94 | 94.29 | 63.80 | 60.9-66.6 | 0.05 | 64.12 | 0.16 |
| 1 | 3 | 94.89 | 93.19 | 63.70 | 60.8-66.5 | 0.05 | 63.79 | 0.26 |
| 1 | 4 | 86.35 | 88.80 | 32.10 | 29.7-34.6 | 0.13 | 0.24 | 0.41 |
| 1 | 5 | 86.18 | 89.02 | 31.60 | 29.2-34.1 | 0.13 | 0.20 | 0.42 |

*Recommended formula (identities/HSP length), which is liberated of genome length and is thus prosperous against the use of complete/draft genome.

**Table 3S** Cholesterol-lowering genes (deconjugation of bile salt and cholesterol assimilation)

| **Gene** | **Gene description** |
| --- | --- |
| Deconjugation of bile salt |  |
| *bsh* | Choloylglycine hydrolase |
| Cholesterol assimilation abilities | |
| ccpA | Catabolite control protein A |
| fba | Class II fructose‐1,6‐bisphosphate aldolase |
| *PYGL* | Glycogen phosphorylase |
| MFS | MFS transporter |

Data obtained from DFast.

**Table 4S** The potential genes associated to various probiotic characteristics from the genome of strain NH2-7C.

| **Putative function** | **Genes** | **Gene product** |
| --- | --- | --- |
| **Modulation of immune system / Acid stress** | *clpB* | Potential immunogenic proteins |
|  | *lspA* | Lipoprotein signal peptidase |
|  | *tuf* | Elongation factor Tu |
| **Nutritional synthesis and several essential processes** | *ccpA* | Catabolite control protein A |
| **Cholesterol-lowering effect / Bile resistance** | *bsh* | Choloylglycine hydrolase |
| **Adhesion or interaction with the host** | *ylcC* | Class A sortase |
|  | *dltD* | D-alanyl-lipoteichoic acid biosynthesis protein DltD |
|  | *dltA* | D-alanylation of LTA |
|  | *lspA* | Lipoprotein signal peptidase |
|  | *tuf* | Elongation factor Tu |
|  | *mtsA* | Manganese ABC transporter substrate-binding protein |
|  | *eno2* | Enolase 2 |
|  | *gapB* | Type I glyceraldehyde-3-phosphate dehydrogenase |
|  | *groS* | Co-chaperonin GroES |
|  | *groL* | Chaperonin GroEL |
|  | *pgi* | Glucose-6-isomerase |
| **Acid stress** | *atpC* | ATP synthase subunit epsilon |
|  | *atpD* | ATP synthase subunit beta |
|  | *atpA* | ATP synthase subunit alpha |
|  | *atpG* | ATP synthase subunit gamma |
|  | *atpH* | ATP synthase subunit delta |
|  | *atpF* | ATP synthase subunit B |
|  | *atpB* | ATP synthase subunit A |
|  | *atpE* | ATP synthase subunit C |
|  | *recA* | Protein RecA (recombinase A) |
|  | *relA* | GTP pyrophosphokinase |
|  | *groS* | Co-chaperonin GroES |
|  | *groL* | Chaperonin GroEL |
|  | *aspS* | Aspartate-tRNA ligase |
| **Acid stress/Bile resistance** | *dnaK* | Chaperone protein DnaK |
|  | *dnaJ* | Chaperone protein DnaJ |
|  | *glmU* | Bifunctional UDP-N acetylglucosamine diphosphorylase/glucosamine phosphate |
|  | *luxS* | S-ribosylhomocysteine lyase |
|  | *gadB* | Glutamate decarboxylase; GABA transporter |
| **Bile resistance** | *nagB* | Glucosamine-6-phosphate deaminase |
|  | *pyrG* | CTP synthase |
|  | *argS* | Arginine-tRNA ligase |
|  | *rpsC* | 30S ribosomal protein S3 |
|  | *rpsE* | 30S ribosomal protein S5 |
|  | *rplD* | 50S ribosomal protein L4 |
|  | *rplE* | 50S ribosomal protein L5 |
|  | *rplF* | 50S ribosomal protein L6 |
| **DNA and protein protection and repair** | *msrB* | Peptide methionine sulfoxide reductase MsrB |
| **Fatty acid synthesis** | *fabD* | Malonyl CoA-acyl carrier protein transacylase |
|  | *fabF* | 3-oxoacyl-[acyl-carrier-protein] synthase II |
|  | *fabI* | Enoyl-[acyl-carrier-protein] reductase [NADH] |
|  | *accC* | acetyl-CoA carboxylase biotin carboxylase subunit |
| **Transcriptional regulator** | *ctsR* | Transcriptional regulator CtsR |
|  | *hrcA* | Heat-inducible transcriptional repressor HrcA |
| **Matabolic rearrangement** | *aldB* | Alpha-acetolactate decarboxylase |

**Supplementary Figures**


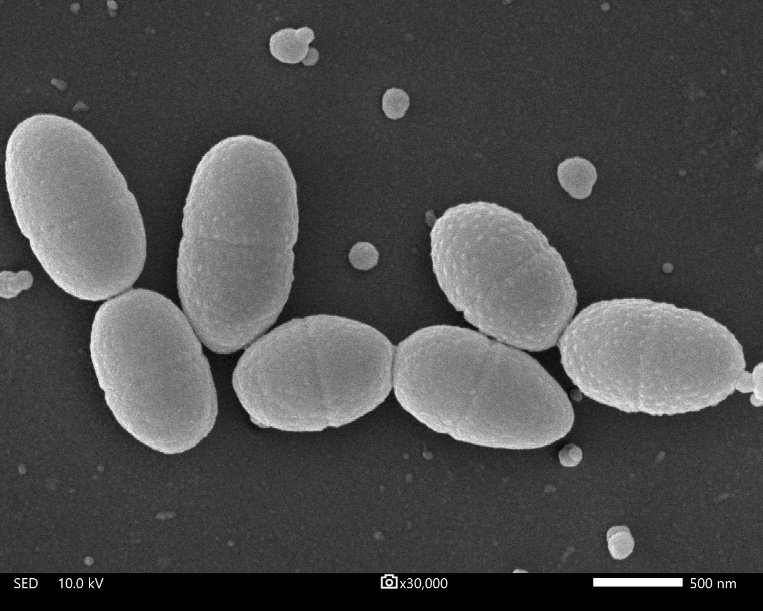


**Figure 1S** Scanning electron micrograph of the strain NH2-7C grown on MRS agar at 30 °C for 3 days.

**
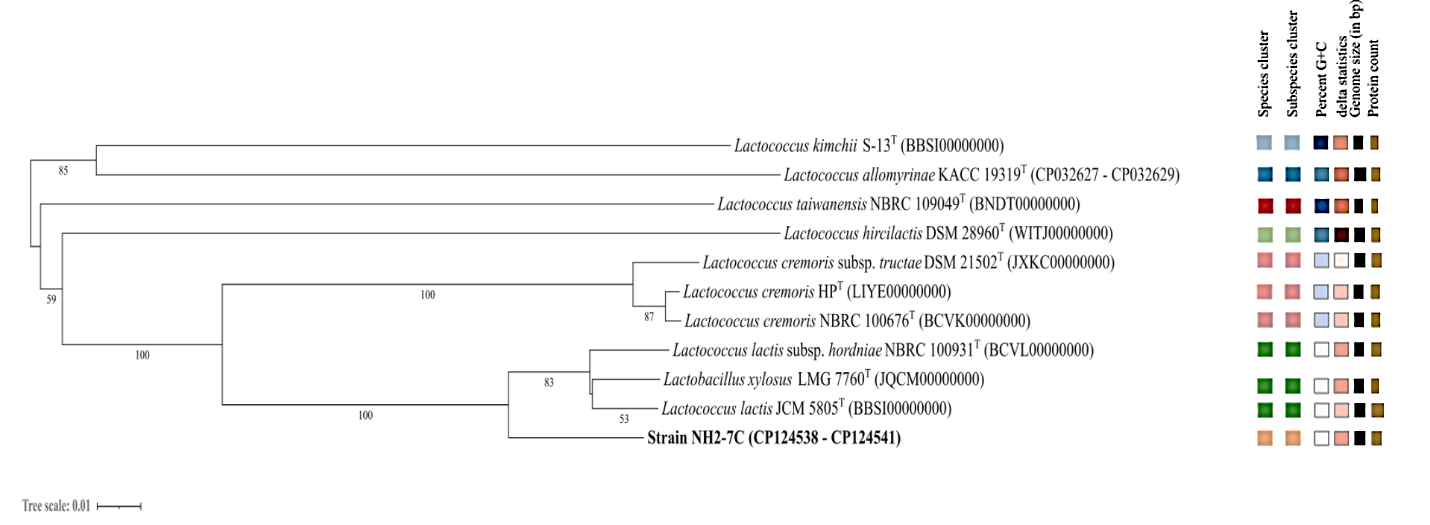
**

**Figure 2S** Phylogenomic tree based on whole genome sequence data result of strain NH2-7C and closely related type strains reconstructed on the Type (Strain) Genome Server (TYGS).

**
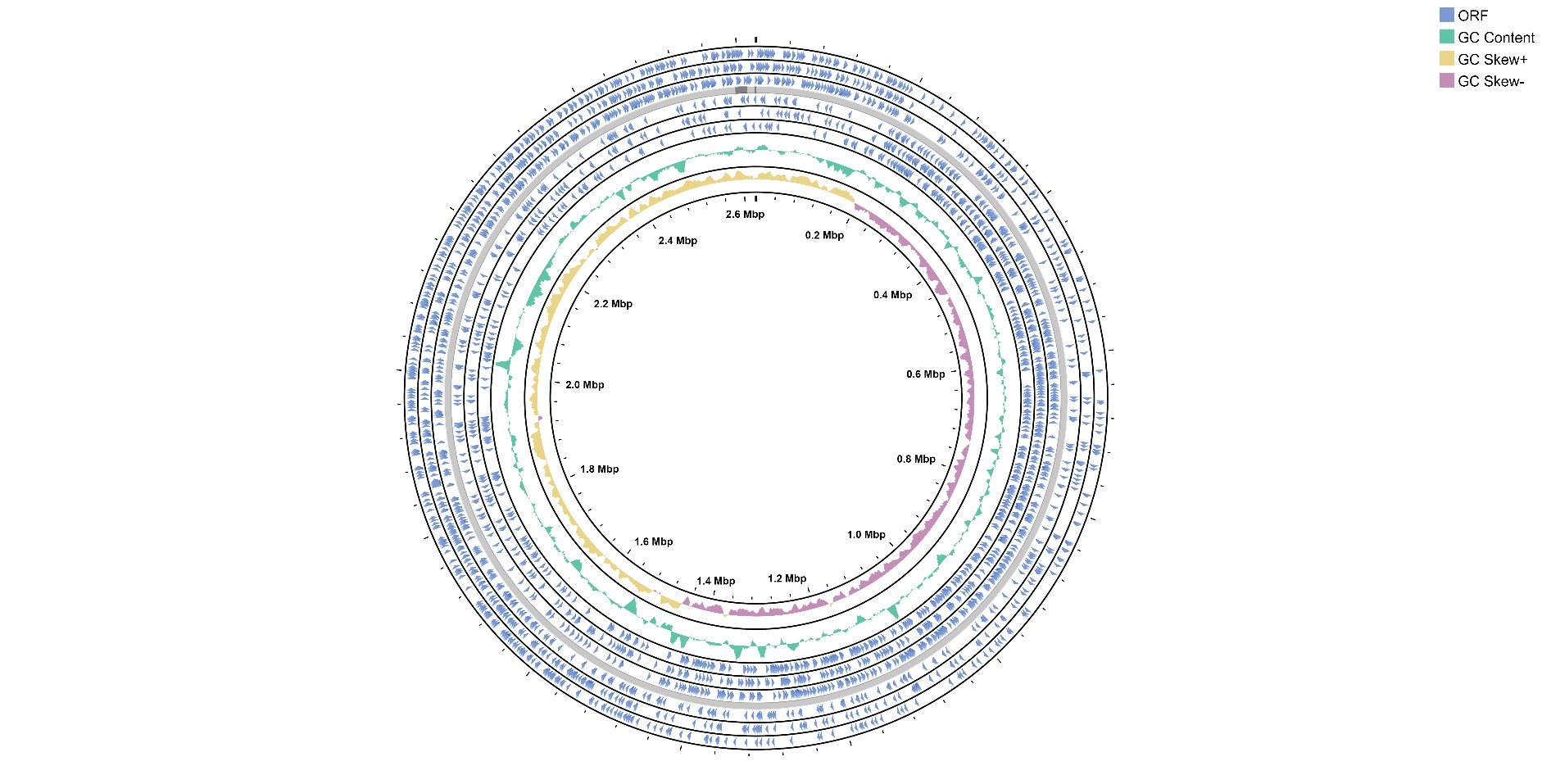
**

**Figure 3S** Circular genome map of *Lactococcus* sp. NH2-7C. The information is indicated as follows: open reading frames (ORFs) (blue), GC skew (+) (yellow), GC skew (−) (pink), and GC content (green).


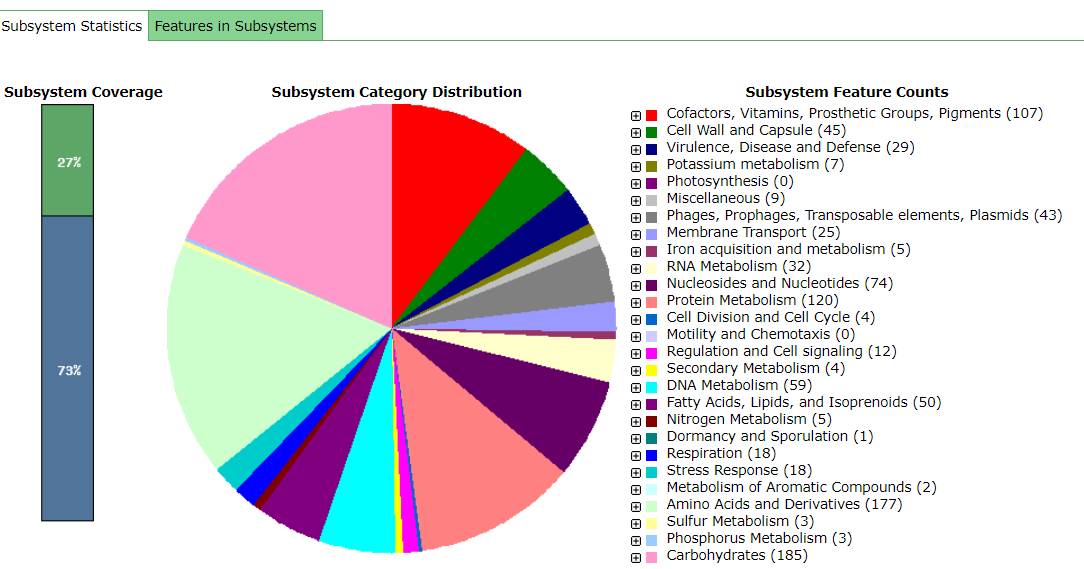


**Figure 4S** The subsystem categories assigned to the genome of strain NH2-7C. The genomic sequence of the strain NH2-7C was annotated using the Rapid Annotation System Technology (RAST) server.


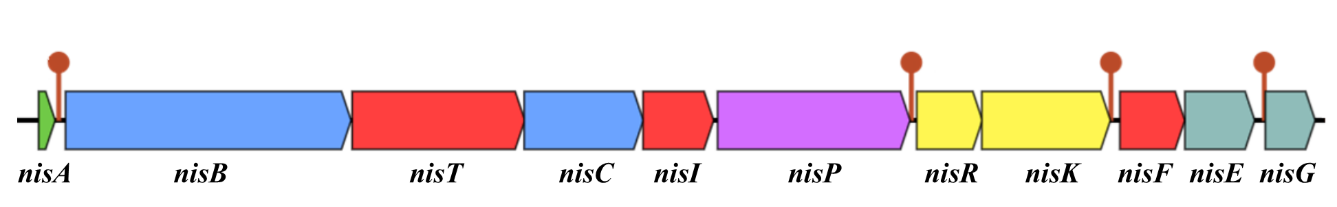


**Figure 5S** The gene cluster encoding the production of nisin A in strain NH2-7C using the online BAGEL v.4.0. web-based tool. Terminators are shown as maroon line circle ends
